# Multiple doses of SHR-1222, a sclerostin monoclonal antibody, in postmenopausal women with osteoporosis: A randomized, double-blind, placebo-controlled, dose-escalation phase 1 trial

**DOI:** 10.3389/fendo.2023.1168757

**Published:** 2023-04-05

**Authors:** Zhijie Dai, Ronghua Zhu, Zhifeng Sheng, Guijun Qin, Xianghang Luo, Qun Qin, Chunli Song, Liping Li, Ping Jin, Guoping Yang, Yanxiang Cheng, Danhong Peng, Chong Zou, Lijuan Wang, Jianzhong Shentu, Qin Zhang, Zhe Zhang, Xiang Yan, Pingfei Fang, Qiangyong Yan, Lingfeng Yang, Xiao Fan, Wei Liu, Bo Wu, Rongrong Cui, Xiyu Wu, Yuting Xie, Chang Shu, Kai Shen, Wenhua Wei, Wei Lu, Hong Chen, Zhiguang Zhou

**Affiliations:** ^1^ National Clinical Research Center for Metabolic Diseases, Key Laboratory of Diabetes Immunology, Ministry of Education, and Department of Metabolism and Endocrinology, The Second Xiangya Hospital of Central South University, Changsha, China; ^2^ Phase I Clinical Trial Center and Department of Pharmacy, The Second Xiangya Hospital of Central South University, Changsha, China; ^3^ National Clinical Research Center for Metabolic Diseases, Hunan Provincial Key Laboratory of Metabolic Bone Diseases, Department of Metabolism and Endocrinology, and Health Management Center, The Second Xiangya Hospital of Central South University, Changsha, China; ^4^ Department of Endocrinology and Metabolism, The First Affiliated Hospital of Zhengzhou University, Zhengzhou, China; ^5^ Department of Endocrinology, Xiangya Hospital of Central South University, Changsha, China; ^6^ National Agency for Clinical Trial of Medicines, Xiangya Hospital of Central South University, Changsha, China; ^7^ Orthopedics Department, Peking University Third Hospital, Beijing, China; ^8^ Endocrine Department, The First Affiliated Hospital of Henan University of Science and Technology, Luoyang, China; ^9^ Department of Endocrinology, The Third Xiangya Hospital of Central South University, Changsha, China; ^10^ Center of Clinical Pharmacology, The Third Xiangya Hospital of Central South University, Changsha, China; ^11^ Department of Gynecology, Renmin Hospital of Wuhan University, Wuhan, China; ^12^ Department of Gynaecology and Obstetrics, Zhongda Hospital Southeast University, Nanjing, China; ^13^ Department of Clinical Pharmacology, Jiangsu Province Hospital of Chinese Medicine, Nanjing, China; ^14^ Department of Endocrinology, Jiangsu Province Hospital of Chinese Medicine, Nanjing, China; ^15^ Clinical Pharmacy, The First Affiliated Hospital of Zhejiang University School of Medicine, Hangzhou, China; ^16^ Department of Geriatrics, The First Affiliated Hospital of Zhejiang University School of Medicine, Hangzhou, China; ^17^ Endocrinology and Metabolism, The First Affiliated Hospital of Zhejiang University School of Medicine, Hangzhou, China; ^18^ Clinical Research & Development, Jiangsu Hengrui Pharmaceuticals Co., Ltd, Shanghai, China

**Keywords:** sclerostin, postmenopausal osteoporosis, pharmacokinetics, pharmacodynamics, bone mineral density

## Abstract

SHR-1222, a novel humanized monoclonal antibody targeting sclerostin, has been shown to induce bone formation and decrease bone resorption at a single dose ranging 50–400 mg in our previous phase 1 trial. This study was a randomized, double-blind, placebo-controlled, dose-escalation phase 1 trial, which further investigated the safety, tolerability, pharmacokinetics (PK), pharmacodynamics (PD), and immunogenicity of multiple ascending doses of SHR-1222 in women with postmenopausal osteoporosis (POP). A total of 105 women with POP were enrolled and randomly assigned. Twenty-one received placebo and eighty-four received SHR-1222 sequentially (100 mg QM, n=4; 200 or 300 mg QM, n=20; and 400 or 600 mg Q2M, n=20). The most common adverse events included increased blood parathyroid hormone, increased low-density lipoprotein, increased blood alkaline phosphatase, increased blood cholesterol, back pain, and arthralgia, the majority of which were mild in severity without noticeable safety concerns. Serum SHR-1222 exposure (C_max,ss_ and AUC_0-tau,ss_) increased in a greater than dose-proportional manner. Following multiple doses of SHR-1222, the bone formation markers (terminal propeptide of type I procollagen, bone-specific alkaline phosphatase, and osteocalcin) increased in a dose-dependent manner, whereas the bone resorption marker (β-C-telopeptide) was downregulated. Accordingly, BMD gains in the lumbar spine, total hip, and femoral neck were observed. The maximum BMD increase from baseline at the lumbar spine was detected in the 300 mg QM cohort (14.6% vs. 0.6% in the placebo group on day 169). Six (6/83; 7.2%) subjects developed anti-SHR-1222 antibodies with no discernible effects on PKs, PDs, and safety. Thus, multiple doses of SHR-1222 showed an acceptable safety profile and dose-dependent plasma exposure in women with POP, and could improve their BMD rapidly and prominently by promoting bone formation and inhibiting bone resorption. These findings further support SHR-1222 as a potential alternative agent for the treatment of POP.

## Introduction

Osteoporosis is defined as a progressive systemic skeletal disease characterized by low bone mass and micro-architectural deterioration of bone tissue, with a consequent increase in bone fragility and susceptibility to fracture by the NIH Consensus Development Panel on Osteoporosis Prevention, Diagnosis, and Therapy (1). There are multiple risk factors for osteoporosis, among which age and sex hormones are the most significant. Postmenopausal women are at great risk of osteoporosis, and rapid bone loss due to estrogen deficiency after the onset of menopause is associated with an increased risk of fracture (2). As previously reported (3), the estimated prevalence of osteoporosis is 32.1% in women > 50 years of age and 51.6% in women > 65 years of age in China. Approximately one in two women aged > 50 years will experience an osteoporotic-related fracture in their lifetime (4).

Medications used to treat osteoporosis are classified as either antiresorptive or anabolic in action (5). Bisphosphonates are the most widely used agents for the prevention and treatment of osteoporosis and promote the apoptosis of osteoclasts actively engaged in the degradation of minerals on the bone surface (6). However, the long-term use of bisphosphonates increases the risk of upper gastrointestinal irritation, as well as complications such as medication-related osteonecrosis of the jaw and atypical femoral fractures. In addition, bisphosphonates reduce both osteoclast and osteoblast activity, which consequently suppresses bone remodeling (7, 8). On the other hand, anabolic drugs, such as parathyroid hormone (PTH) and parathyroid hormone-related protein (PTHrP) analogs (for example, teriparatide and abaloparatide) can stimulate bone formation and enhance bone remodeling (9). Teriparatide has been shown to enhance bone density by stimulating the formation and action of osteoblasts and promoting bone formation in patients with osteoporosis. However, treatment with teriparatide is usually limited to a lifetime maximum duration of 2 years based on the possible risk of osteosarcoma. Additional treatment or retreatment with teriparatide beyond 2 years should only be considered for individuals who remain at or return to having a high risk for fracture (10).

Mechanistically, as a negative regulator of the Wnt signaling pathway, sclerostin binds low-density lipoprotein receptor-related protein 5/6 (LRP5/6) co-receptors, further inhibiting bone formation and promoting bone resorption (11, 12). Sclerostin may also act as an enhancer of osteoclastogenesis by upregulating the synthesis of the receptor activator of NF-κB ligand (13). Therefore, anti-sclerostin agents, such as romosozumab and blosozumab, are considered an attractive treatment option, which results in enhanced BMD, improved bone structure, and increased bone strength (14). Prior phase 3 trials (FRAME and ARCH studies) demonstrated that a significantly lower risk of new vertebral fracture was observed in women with postmenopausal osteoporosis (POP) treated with romosozumab than in those treated with placebo or alendronate (15, 16).

Romosozumab is the only humanized therapeutic antibody against sclerostin that has been authorized for use in the United States, the European Union, Japan, Korea, and Canada. However, a safety warning for romosozumab for the risk of cardiovascular (CV) events (myocardial infarction, stroke, and cardiovascular death) should be noted (17, 18). Moreover, the duration of romosozumab use should be limited to 12 monthly doses since the anabolic effect of romosozumab wanes after 12 monthly doses. If osteoporosis therapy remains warranted, continued therapy with an antiresorptive agent should be considered (17). It is reported that safety observations did not influence the decision to limit romosozumab treatment to 12 months (19). However, considering its high risk of CV events, it seems reasonable to limit use until more evidence is available. These restrict the clinical application of romosozumab. Blosozumab, another anti-sclerostin agent previously studied, has completed phase 1 and phase 2 trials, showing increased BMD in a dose-dependent manner (20, 21). However, its phase 3 results are still awaited. Thus, there is still a need to develop safer alternative sclerostin inhibitors.

SHR-1222 is a novel humanized monoclonal antibody targeting sclerostin that was developed for the treatment of osteoporosis. Preclinical studies have demonstrated that SHR-1222 exhibits high affinity for human sclerostin (data on file, Jiangsu Hengrui Pharmaceuticals). Moreover, our first-in-human clinical trial (NCT03870100) indicated that single doses of SHR-1222 ranging 50–400 mg were generally well tolerated and promoted bone formation, inhibited bone resorption, and increased BMD in healthy men and postmenopausal women with low bone mass (22). Here, we conducted a phase 1 trial to evaluate the safety, tolerability, pharmacokinetics (PKs), pharmacodynamics (PDs), and immunogenicity of 24-week SHR-1222 treatment with multiple doses in women with POP.

## Methods

### Study design

This was a randomized, double-blind, placebo-controlled, dose-escalation phase 1 trial of SHR-1222 in women with POP conducted at 11 sites in China (NCT04435158). Subject participation consisted of a 30-d screening period, 24-week treatment period, and 57-d treatment-free follow-up period.

The study was conducted in accordance with the Declaration of Helsinki and the International Conference on Harmonization/Good Clinical Practice Guidelines as well as local regulatory requirements. The study protocol and amendments were approved by the ethics committees of all participating centers, and all subjects provided written informed consent before enrolment. This study was registered with ClinicalTrials.gov (NCT04435158).

### Study participants

Eligible subjects were ambulatory women of postmenopausal status for at least 5 years, aged 50–70 years, with evidence of osteoporosis and body weight of no less than 40 kg. Postmenopausal status was defined as having ≥ 12 months of spontaneous amenorrhea or undergoing bilateral oophorectomy 6 weeks prior. Osteoporosis was defined as a BMD T-score ≤ -2.5 at the lumbar spine (L1–L4, one or more vertebrae), total hip, or femoral neck using the Asian normative database integrated with the Hologic dual energy X-ray absorptiometry (DXA) scanner. The subjects had to have at least two vertebrae in the L1–L4 region and at least one hip that could be evaluated using dual-energy X-ray absorptiometry. Key exclusion criteria included a history of hip fracture, any severe or more than two moderate vertebral fractures, a history of metabolic bone diseases (except for osteoporosis) or endocrine and metabolic diseases that may disturb bone metabolism, a T-score < -3.5, at lumbar spines (L1–L4, one or more vertebrae), total hip or femoral neck, vitamin D deficiency (25-hydroxyvitamin D [25-OHD] concentration < 20 ng/mL), diabetes inadequately controlled on diet and exercise (fasting plasma glucose ≥ 7.0 mmol/L or hemoglobin A1c ≥ 7.0%), and hypercalcemia or hypocalcemia. The use of the following agents was not allowed: intravenous bisphosphonates or denosumab at any time, oral bisphosphonates, PTH or its analogs, strontium or fluoride within the previous 12 months, long-acting estrogen/progesterone replacement therapy within the previous 6 months, systemic glucocorticoids, estrogen/progesterone replacement therapy, anabolic steroids, selective estrogen-receptor modulators, calcitonin, calcitriol and its analogs, and thiazide diuretics within the previous 3 months.

### Treatment interventions

**Figure 1:**
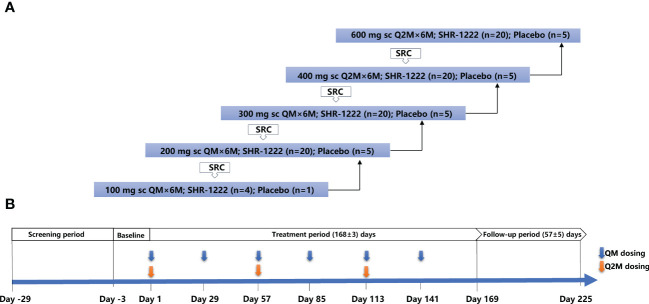
Study design. **(A)** Framework for the dose-escalation of SHR-1222. **(B)** Dosing and assessment schema. QM, once every month; Q2M, once every 2 months; SRC, safety review committee.

Women with POP received SHR-1222 or a matching placebo subcutaneously at doses of 100, 200, or 300 mg once monthly (QM) and 400 or 600 mg once every 2 months (Q2M) sequentially (**Figure 1**). If one subject completed randomization but withdrew from the trial before the first dosing of the study treatment, a successor was enrolled. In the 100 mg QM group, five subjects were randomized, four received SHR-1222 and one received placebo. In the 200 mg QM, 300 mg QM, 400 mg Q2M and 600 mg Q2M groups, 102 subjects were randomly assigned, of whom 80 received SHR-1222, 20 received placebo, and two withdrew.

The decision to proceed to the next dosing cohort was made by the safety review committee (SRC) consisting of sponsor representatives and investigators after all subjects in the previous cohort received the study treatment and had been monitored for no less than 36 days after dosing. Treatment was administered to each cohort for a total of 24 weeks (six doses for QM groups or three doses for Q2M groups).

For subjects whose baseline serum 25-OHD level was 20–30 ng/mL, a loading dose of 400,000 IU of vitamin D was administered at baseline. All subjects were given daily calcium (500–600 mg) and vitamin D_2_ or D_3_ (800–1200 IU) throughout the study period.

### Endpoints and assessments

The primary endpoint of this trial was the assessment of the safety and tolerability of multiple subcutaneous injections of SHR-1222. The secondary endpoints included PKs, PDs, and the immunogenicity of SHR-1222.

Adverse events (AEs), coded according to the preferred terms of the Medical Dictionary for Regulatory Activities v25.0, were monitored and recorded. Clinical laboratory evaluations, vital signs, and 12-lead electrocardiograms were conducted for safety assessments.

Blood samples for PKs and PDs were collected in the morning after an overnight fast for at least 10 hours at 19 time points: days 1, 3, 7, 15, 22, 29, 57, 85, 113, 141 (at predose if dosing); the day after the last dose of the study treatment for 2, 4, 6, 14, or 21 d (days 115, 117, 119, 127, 134 for Q2M cohorts; days 143, 145, 147, 155, and 162 for QM cohorts); and days 169, 197, and 225. PK analyses were performed at Shanghai InnoStar Bio-tech Co., Ltd., including area under the curve from time 0 to the last measurable concentration (AUC_0-t_), area under the concentration-time curve from zero to time infinity (AUC_0-inf_), time to reach maximum serum concentration (T_max_), maximum serum concentration (C_max_), average serum concentration (C_avg_), half-life (t_1/2_), apparent total clearance (CL/F), apparent volume of distribution (V_z_/F), area under the curve between dose intervals (AUC_0-tau_), trough plasma concentration (C_trough_), accumulation index (R_acc_), and degree of fluctuation (DF). Pharmacodynamic indices included bone formation markers of terminal propeptide of type I procollagen (P1NP), bone-specific alkaline phosphatase (BSAP), osteocalcin (OST), and the bone resorption marker β-C-telopeptide (β-CTx), which were analyzed at a central laboratory (Guangzhou Kingmed Center for Clinical Laboratory Co.,Ltd.) using a chemiluminescence method (Roche Diagnostics Test Kits for P1NP, OST, and β-CTx; Beckman Coulter Diagnostics Test Kits for BSAP).

All subjects underwent lumbar spine (L1–L4) and hip (total hip and femoral neck) DXA scans for BMD measurements during the screening period, and on days 57, 85, 169, 197, and 225, using the same Hologic Discovery Wi Bone Densitometer (Hologic, Bedford, MA, United States) at each center. Instrument quality controls were produced to ensure the coefficient of variation (CV) under 1.5% throughout the study to ensure the accuracy of the results.

Blood samples for anti-SHR-1222 antibody determination were collected on days 1, 7, 29, 57, 85, 113, 141 (at pre-dose if dosing), 6 d after the last dose of study treatment (days 119 and 147 for Q2M and QM cohorts, respectively), and on day 225. Anti-SHR-1222 antibody was detected at Shanghai InnoStar Bio-tech Co., Ltd. using a validated Meso Scale Discovery electrochemiluminescence immunoassay. Blood samples were stored or transported at ultra-low temperatures ranging from -100°C to -60°C and analyzed in batches.

### Statistical analysis

All subjects who received placebo were included in the placebo group. Safety was assessed in all subjects who received at least one dose of SHR-1222 or a placebo. PK concentration and parameter analyses were performed in subjects who received at least one dose of SHR-1222, with at least one qualified PK concentration/parameter result. PD indicators were analyzed in those who received at least one dose of SHR-1222 or a placebo, with no missing baseline and at least one qualified post-baseline PD assessment data. Immunogenicity was assessed in patients who received at least one dose of SHR-1222 or a placebo, with baseline and at least one post-baseline qualified sample.

The sample size was estimated based on the percentage changes from baseline to the 6^th^ month in BMD at the lumbar spine at SHR-1222 doses of 200 mg QM, 300 mg QM, 400 mg Q2M and 600 mg Q2M. Assuming a standard deviation (SD) of 5%, α=0.05 (two-sided), the tolerable probability is 0.8, and the allocation ratio between the SHR-1222 group and the placebo group is 1:1 (20:20), then 40 subjects (each SHR-1222 group, n=20; placebo group, n=20) can provide a 95% confidence interval (CI) for the treatment difference between the SHR-1222 dosing group (200 mg QM, 300 mg QM, 400 mg Q2M or 600 mg Q2M) and the placebo group with a half interval width around 3.5%. In addition, 5–10 subjects may have been recruited in the SHR-1222 100 mg QM cohort, with 4–8 subjects treated with SHR-1222 and the remaining treated with placebo. Thus, in total, approximately 105–110 subjects were needed in this study, including 4–8 in the SHR-1222 100 mg QM group, 20 in each of the SHR-1222 200 mg QM, 300 mg QM, 400 mg Q2M and 600 mg Q2M groups, and 21–22 in the placebo group.

Serum SHR-1222 concentration-time data were analyzed using non-compartmental methods. Baseline demographics, safety results, and PK parameters by treatment and dose were tabulated and summarized using descriptive statistics. At every scheduled visit, the percentage changes from the baseline for selected PD parameters and BMD were calculated. All statistical analyses were performed using SAS, version 9.4 (SAS Institute, Inc.).

## Results

### Study participants

Between September 23, 2020, and November 29, 2021, 107 subjects who were eligible underwent randomization to receive different dosing regimens of SHR-1222 or matching placebo. Two subjects in the placebo group, who did not receive the assigned treatment, withdrew from the study. Of the 105 subjects, 21 received placebo and 84 received SHR-1222 (100 mg QM, n=4; 200 mg QM, 300 mg QM, 400 mg Q2M or 600 mg Q2M, n=20 each). Ninety-nine subjects completed the preplanned treatment period, and six stopped early (four due to subject withdrawal [200 mg QM, n=2; 300 mg QM, n=1; and 400 mg Q2M, n=1], one due to consent withdrawal in the 600 mg Q2M group, and one due to other reasons in the 300 mg Q2M group).

The baseline demographics and clinical characteristics were similar between the placebo and SHR-1222 treatment groups (**Table 1**). The mean age of the patients in each cohort ranged 58.6–60.0 years. At baseline, the mean DXA T-score of each group ranged from -2.98 to -2.56 at the lumbar spine, -1.93 to -1.60 at the total hip, and -2.56 to -2.25 at the femoral neck, respectively. The mean 25-OHD level was 39.2 ng/mL in the placebo group and ranged 23.6–38.7 ng/mL across the SHR-1222 groups.

**Table 1:** Demographics and baseline characteristics.

	Pooled Placebo (N=21)	SHR-1222					
		100mg QM (N=4)	200mg QM (N=20)	300mg QM (N=20)	400mg Q2M (N=20)	600mg Q2M (N=20)	Total (N=84)
Age (years)							
Mean ± SD	59.4 ± 4.5	60.0 ± 7.1	58.6 ± 5.0	58.6 ± 5.1	59.7 ± 4.1	58.8 ± 5.9	58.9 ± 5.1
Median (range)	59.0 (51–67)	60.5 (51-68)	56.5 (51-68)	58.0 (50-67)	59.0 (53-69)	59.0 (51-69)	59.0 (50-69)
BMI, kg/m^2^, mean ± SD	22.61 ± 2.75	20.82 ± 1.19	23.99 ± 2.40	22.16 ± 2.90	23.81 ± 2.15	23.65 ± 2.00	23.28 ± 2.47
BMD T score, mean ± SD							
Lumbar spine L1-L4	-2.56 ± 0.63	-2.98 ± 0.64	-2.70 ± 0.54	-2.97 ± 0.38	-2.57 ± 0.66	-2.68 ± 0.67	-2.74 ± 0.58
Total hip	-1.75 ± 0.44	-1.93 ± 0.58	-1.60 ± 0.54	-1.80 ± 0.55	-1.91 ± 0.57	-1.83 ± 0.69	-1.79 ± 0.59
Femoral neck	-2.25 ± 0.61	-2.40 ± 0.57	-2.31 ± 0.56	-2.29 ± 0.56	-2.56 ± 0.48	-2.45 ± 0.65	-2.40 ± 0.56
Biomarkers, mean ± SD							
P1NP (ng/ml)	72.41 ± 21.77	73.23 ± 18.00	66.47 ± 26.10	77.81 ± 23.01	71.29 ± 26.45	70.53 ± 20.71	71.61 ± 23.70
BSAP (μg/L)	15.34 ± 4.13	13.94 ± 5.40	16.63 ± 5.50	16.09 ± 4.70	14.99 ± 4.12	15.72 ± 5.30	15.78 ± 4.87
OST (ng/ml)	22.69 ± 7.47	20.38 ± 5.30	21.25 ± 6.57	24.75 ± 8.27	24.40 ± 8.59	21.18 ± 6.40	22.75 ± 7.46
β-CTx (ng/ml)	0.52 ± 0.20	0.59 ± 0.18	0.58 ± 0.22	0.59 ± 0.20	0.51 ± 0.26	0.44 ± 0.17	0.53 ± 0.22
eGFR (ml/min1.73m^2^), mean ± SD	88.1 ± 15.4	102.5 ± 14.7	105.7 ± 26.7	92.9 ± 15.9	88.3 ± 14.8	91.3 ± 17.2	93.6 ± 19.1
25-OHD (ng/ml), mean ± SD	39.2 ± 16.7	23.6 ± 16.1	31.5 ± 13.0	34.0 ± 13.6	31.3 ± 12.1	38.7 ± 18.5	33.4± 14.7

eGFR was calculated using the modified MDRD formula for Chinese: eGFR (ml/min1.73m^2^) =186×(Scr)^-1.154^×(Age)^-0.203^×(0.742 if female).

QM, once every month; Q2M, once every 2 months; SD, standard deviation; BMI, body-mass index; BMD, bone mineral density. P1NP, N-terminal propeptide of type 1 procollagen; BSAP, bone-specific alkaline phosphatase; OST, osteocalcin; β-CTx, β-C-telopeptide; Scr, serum creatinine; eGFR, estimated glomerular filtration rate; 25-OHD, 25-hydroxyvitamin D.

### Safety

A total of 100 subjects experienced at least one AE, including 81 (96.4%) in the SHR-1222 group and 19 (90.5%) in the placebo group (**Table 2**). Most AEs were mild in severity. Only 14.3% (3/21) of the subjects treated with placebo and 17.9% (15/84) of the subjects who received SHR-1222 had moderate AEs. No severe AEs or AEs leading to withdrawal, treatment interruption, discontinuation, or death were reported. Only one subject treated with placebo experienced a serious AE (thoracic vertebral fracture-caused hospitalization), which was judged to be unrelated to the study treatment by the investigator. The most common AEs that occurred at least 5% higher in subjects treated with SHR-1222 than in those treated with placebo mainly included increased blood PTH (41.7% vs. 9.5%), increased low-density lipoprotein (LDL; 14.3% vs. 0), increased blood alkaline phosphatase (ALP; 13.1% vs. 0), increased blood cholesterol (11.9% vs. 4.8%), back pain (10.7% vs. 4.8%), and arthralgia (6.0% vs. 0). Except for increased blood ALP (0, 10.0%, 25.0%, 0, and 20.0% in the 100 mg QM, 200 mg QM, 300 mg QM, 400 mg Q2M and 600 mg Q2M group, respectively), no other dose-dependent trend was observed among the AEs. The frequency rate of increased CK-MB (6.0% vs. 9.5%) was comparable between the SHR-1222 and placebo groups.

**Table 2:** Adverse events in the safety set.

	Pooled Placebo (N=21)	SHR-1222					
		100 mg QM (N=4)	200 mg QM (N=20)	300 mg QM (N=20)	400 mg Q2M (N=20)	600 mg Q2M (N=20)	Total (N=84)
Any AEs	19 (90.5)	3 (75.0)	20 (100.0)	18 (90.0)	20 (100.0)	20 (100.0)	81 (96.4)
Mild	19 (90.5)	3 (75.0)	18 (90.0)	18 (90.0)	20 (100.0)	20 (100.0)	79 (94.0)
Moderate	3 (14.3)	1 (25.0)	5 (25.0)	6 (30.0)	3 (15.0)	0	15 (17.9)
Severe	0	0	0	0	0	0	0
Treatment-related AEs	13 (61.9)	3 (75.0)	17 (85.0)	16 (80.0)	16 (80.0)	12 (60.0)	64 (76.2)
Serious AEs	1 (4.8)	0	0	0	0	0	0
AEs occurring at least 5% of patients assigned to either SHR-1222 or placebo							
Blood parathyroid hormone increased ^a^	2 (9.5)	2 (50.0)	5 (25.0)	9 (45.0)	8 (40.0)	11 (55.0)	35 (41.7)
Hyperlipidemia ^b^	6 (28.6)	1 (25.0)	5 (25.0)	7 (35.0)	6 (30.0)	6 (30.0)	25 (29.8)
Blood triglycerides increased ^b^	4 (19.0)	1 (25.0)	8 (40.0)	4 (20.0)	3 (15.0)	1 (5.0)	17 (20.2)
White blood cells urine positive	3 (14.3)	1 (25.0)	3 (15.0)	4 (20.0)	3 (15.0)	4 (20.0)	15 (17.9)
Low density lipoprotein increased ^ab^	0	3 (75.0)	1 (5.0)	5 (25.0)	2 (10.0)	1 (5.0)	12 (14.3)
Urine leukocyte esterase positive	3 (14.3)	2 (50.0)	1 (5.0)	3 (15.0)	3 (15.0)	2 (10.0)	11 (13.1)
Blood alkaline phosphatase increased ^a^	0	0	2 (10.0)	5 (25.0)	0	4 (20.0)	11 (13.1)
Blood cholesterol increased ^ab^	1 (4.8)	0	1 (5.0)	3 (15.0)	3 (15.0)	3 (15.0)	10 (11.9)
Upper respiratory tract infection	4 (19.0)	1 (25.0)	4 (20.0)	1 (5.0)	1 (5.0)	2 (10.0)	9 (10.7)
Urinary tract infection	2 (9.5)	1 (25.0)	2 (10.0)	1 (5.0)	2 (10.0)	3 (15.0)	9 (10.7)
Back pain ^a^	1 (4.8)	0	0	3 (15.0)	4 (20.0)	2 (10.0)	9 (10.7)
Blood 25-hydroxycholecalciferol decreased	2 (9.5)	0	1 (5.0)	3 (15.0)	1 (5.0)	3 (15.0)	8 (9.5)
Asthenia	1 (4.8)	0	1 (5.0)	2 (10.0)	4 (20.0)	0	7 (8.3)
White blood cell count decreased	2 (9.5)	0	2 (10.0)	2 (10.0)	1 (5.0)	1 (5.0)	6 (7.1)
Blood calcium increased	2 (9.5)	0	0	1 (5.0)	4 (20.0)	1 (5.0)	6 (7.1)
Urinary occult blood positive	4 (19.0)	0	1 (5.0)	3 (15.0)	0	1 (5.0)	5 (6.0)
Blood creatine phosphokinase MB increased ^b^	2 (9.5)	0	3 (15.0)	1 (5.0)	1 (5.0)	0	5 (6.0)
Electrocardiogram T wave abnormal	1 (4.8)	0	0	1 (5.0)	2 (10.0)	2 (10.0)	5 (6.0)
Blood parathyroid hormone decreased	1 (4.8)	0	2 (10.0)	1 (5.0)	2 (10.0)	0	5 (6.0)
Vitamin D deficiency	1 (4.8)	0	3 (15.0)	1 (5.0)	1 (5.0)	0	5 (6.0)
Dizziness	1 (4.8)	1 (25.0)	1 (5.0)	0	3 (15.0)	0	5 (6.0)
Arthralgia ^a^	0	0	2 (10.0)	2 (10.0)	1 (5.0)	0	5 (6.0)
Hypothyroidism	3 (14.3)	0	1 (5.0)	1 (5.0)	0	2 (10.0)	4 (4.8)
Blood glucose increased	2 (9.5)	0	1 (5.0)	0	2 (10.0)	1 (5.0)	4 (4.8)
Pain in extremity	2 (9.5)	0	0	2 (10.0)	1 (5.0)	0	3 (3.6)
Eosinophil count increased	2 (9.5)	0	0	0	0	0	0
Other prespecified AESI							
Injection site hemorrhage	0	0	1 (5.0)	1 (5.0)	0	1 (5.0)	3 (3.6)
Blood calcium decreased	0	2 (50.0)	0	0	0	0	2 (2.4)
Hypocalcemia	0	0	1 (5.0)	0	0	0	1 (1.2)
Blood phosphorus decreased	0	1 (25.0)	0	0	0	0	1 (1.2)
Hypophosphatemia	0	0	1 (5.0)	0	0	0	1 (1.2)
Lacunar infarction	0	0	0	0	0	1 (5.0)	1 (1.2)

a AEs occurring more frequently in the SHR-1222 group with a difference of ≥5% versus pooled placebo.

b AEs were also predefined as AESI.

AE, adverse event; QM, once every month; Q2M, once every 2 months; AESI, adverse event of special interest.

Systemic allergic reactions (such as anaphylactic shock), local reactions at the injection site, decreased blood calcium, decreased blood phosphorus, increased blood triglycerides, increased blood cholesterol, and cardiovascular and cerebrovascular ischemic events were prespecified adverse events of special interest (AESIs). Injection site hemorrhage was reported in 3 (3.6%) subjects in the SHR-1222 group and none in the placebo group. Calcium and phosphate metabolic disorders were found in only 4 subjects who received SHR-1222 (decreased blood calcium, 2.4% [n=2]; hypocalcemia, 1.2% [n=1]; decreased blood phosphorus, 1.2% [n=1]; hypophosphatemia, 1.2% [n=1]), but not in the placebo group. AEs of increased blood triglyceride levels occurred in 20.2% (n=17) of subjects with SHR-1222, which was similar to that reported in the placebo group (19.0%, n=4). However, increased blood cholesterol levels were reported in 10 (11.9%) subjects in the SHR-1222 group, which was higher than that observed in the placebo group (4.8%, n=1). One subject in the SHR-1222 600 mg Q2M group experienced an asymptomatic lacunar infarction, which was only noted upon imaging, and the investigator judged the causality as unrelated to the study treatment. Most AESIs are mild in severity and can be self-resolved without special medical intervention.

### PKs

**Figure 2:**
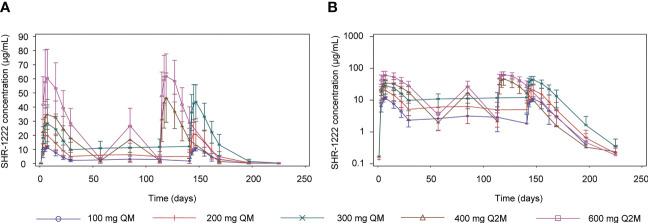
Mean serum concentration-time profiles of SHR-1222. **(A)** Linear plots. **(B)** Semi-log plots. SE, standard error; QM, once every month; Q2M, once every 2 months.

The PK parameters are summarized in **Table 3** and the plasma concentration-time curves are shown in **Figure 2**.

**Table 3:** Pharmacokinetics of SHR-1222: noncompartmental parameter estimates.

		SHR-1222				
		100mg QM (N=4)	200mg QM (N=20)	300mg QM (N=20)	400mg Q2M (N=20)	600mg Q2M (N=20)
At first dose						
T_max_ (day)	Median (Min, Max)	5.94 (3.95, 5.96)	5.92 (3.90, 11.91)	5.96 (3.89, 13.99)	5.98 (3.84, 19.98)	5.90 (3.92, 6.00)
C_max_ (μg/mL)	GeoMean (%CV)	11.7 (3.9%)	19.5 (39.4%)	26.8 (44.6%)	35.0 (32.7%)	58.8 (34.4%)
t_1/2_ (day)	GeoMean (%CV)	7.77 (15.1%)	8.53 (22.6%)	9.34 (16.4%)	9.05 (20.5%)	9.51 (21.0%)
AUC_0-t_ (day·μg/mL)	GeoMean (%CV)	186 (15.3%)	328 (37.1%)	509 (38.6%)	890 (35.3%)	1550 (33.7%)
AUC_0−inf_, (day*μg/mL)	GeoMean (%CV)	212 (19.8%)	392 (41.2%)	593 (39.7%)	914 (37.5%)	1590 (34.6%)
CL/F (L/day)	GeoMean (%CV)	0.471 (20.3%)	0.510 (43.7%)	0.506 (71.2%)	0.438 (51.6%)	0.376 (28.1%)
V_z_/F (L)	GeoMean (%CV)	5.28 (6.4%)	6.28 (38.0%)	6.83 (71.7%)	5.71 (52.3%)	5.07 (23.1%)
At last dose						
T_max,ss_ (day)	Median (Min, Max)	5.92 (5.89, 5.95)	4.00 (1.94, 5.99)	5.88 (3.88, 6.02)	5.94 (3.88, 13.94)	5.98 (3.91, 13.95)
C_max,ss_ (μg/mL)	GeoMean (%CV)	10.7 (16.9%)	19.7 (46.6%)	43.0 (28.5%)	43.8 (39.0%)	62.4 (25.3%)
C_avg,ss_ (μg/mL)	GeoMean (%CV)	6.30 (23.8%)	11.8 (52.3%)	28.3 (29.7%)	17.6 (38.8%)	25.9 (22.6%)
C_trough,ss_ (μg/mL)	GeoMean (%CV)	2.47 (64.9%)	4.01 (71.6%)	10.6 (47.8%)	1.67 (82.4%)	2.02 (59.7%)
t_1/2,ss_ (day)	GeoMean (%CV)	10.2 (12.7%)	10.3 (28.0%)	9.90 (14.6%)	9.59 (35.2%)	10.5 (24.6%)
AUC_0-tau,ss_ (day·μg/mL)	GeoMean (%CV)	176 (32.8%)	331 (52.3%)	791 (29.7%)	988 (38.8%)	1450 (22.6%)
CL_ss_/F (L/day)	GeoMean (%CV)	0.567 (32.1%)	0.604 (50.6%)	0.379 (34.2%)	0.405 (44.8%)	0.414 (20.4%)
V_z,ss_/F (L)	GeoMean (%CV)	8.37 (43.7%)	8.97 (61.2%)	5.42 (35.1%)	5.48 (64.9%)	6.23 (24.5%)
R_acc_C_max_	GeoMean (%CV)	0.917 (13.6%)	0.990 (30.5%)	1.35 (60.0%)	1.24 (22.9%)	1.05 (20.3%)
R_acc_AUC	GeoMean (%CV)	0.947 (20.1%)	1.02 (26.8%)	1.51 (58.3%)	1.12 (21.2%)	1.00 (19.7%)
DF (%)	GeoMean (%CV)	143 (28.8%)	127 (29.9%)	104 (28.1%)	238 (16.9%)	218 (15.9%)

Data are median (range) for T_max_ and geometric mean (CV%) for other parameters.

SD, standard deviation; CV, coefficient of variation; T_max_, time to reach maximum serum concentration; C_max_, maximum serum concentration; C_avg_, average serum concentration; AUC_0-t_, area under the curve from time 0 to the last measurable concentration; AUC_0−inf_, area under the concentration−time curve from zero to time infinity; SS, steady state; AUC_0-tau,ss_, area under the curve between dose interval; t_1/2_, half-life; CL/F, apparent total clearance; V_z_/F, apparent volume of distribution; C_trough_, trough concentration; R_acc,_ accumulation index; R_acc_AUC, accumulation ratio for AUC; DF, degree of fluctuation.

After a single dose of SHR-1222, the median T_max_ ranged 5.90–5.98 d, the geometric mean of half-life (t_1/2_) ranged 7.77–9.51 d, and no dose-dependent manner was observed. The C_max_ was remarkably increased with the ascending dose (geometric mean, 11.7 μg/mL at 100 mg QM, 19.5 μg/mL at 200 mg QM, 26.8 μg/mL at 300 mg QM, 35.0 μg/mL at 400 mg Q2M, and 58.8 μg/mL at 600 mg Q2M, respectively). AUC_0-t_ (186–1550 d·μg/mL) and AUC_0−inf_ (212–1590 d·μg/mL) showed similar trends. No differences were observed in the geometric mean CL/F (0.376–0.510 L/d) and V_z_/F (5.07–6.83 L).

After the last dosing of SHR-1222, the median T_max,ss_ was 4.00–5.98 d, and the geometric mean of t_1/2,ss_ was 9.59–10.5 d. With the same administration schedule, the geometric mean C_max,ss_ (ranging 10.7–43.0 μg/mL in QM groups; 43.8–62.4 in Q2M groups), AUC_0-tau,ss_ (ranging 176–791 d·μg/mL in QM groups; 988–1450 d·μg/mL in Q2M groups), and C_trough,ss_ (ranging 2.47–10.60 μg/mL in QM groups; 1.67–2.02 μg/mL in Q2M groups) were remarkably increased in dose-dependent manners. The R_acc_C_max_ was comparable with SHR-1222 treatment at different dosing regimens, except for 300 mg QM (geometric mean, 1.35 with 300 mg QM, 0.917 with 100 mg QM and 0.990 with 200 mg QM, and 1.24 with 400 mg Q2M and 1.05 with 600 mg Q2M), and R_acc_AUC showed a similar trend. No obvious differences were observed in the geometric mean CL_ss_/F (0.379–0.604 L/d) or V_z,ss_/F (5.42–8.97 L). DF was more distinct in the Q2M groups (geometric mean, 104–143%) than in the QM groups (geometric mean, 218–238%).

### Bone turnover markers

**Figure 3:**
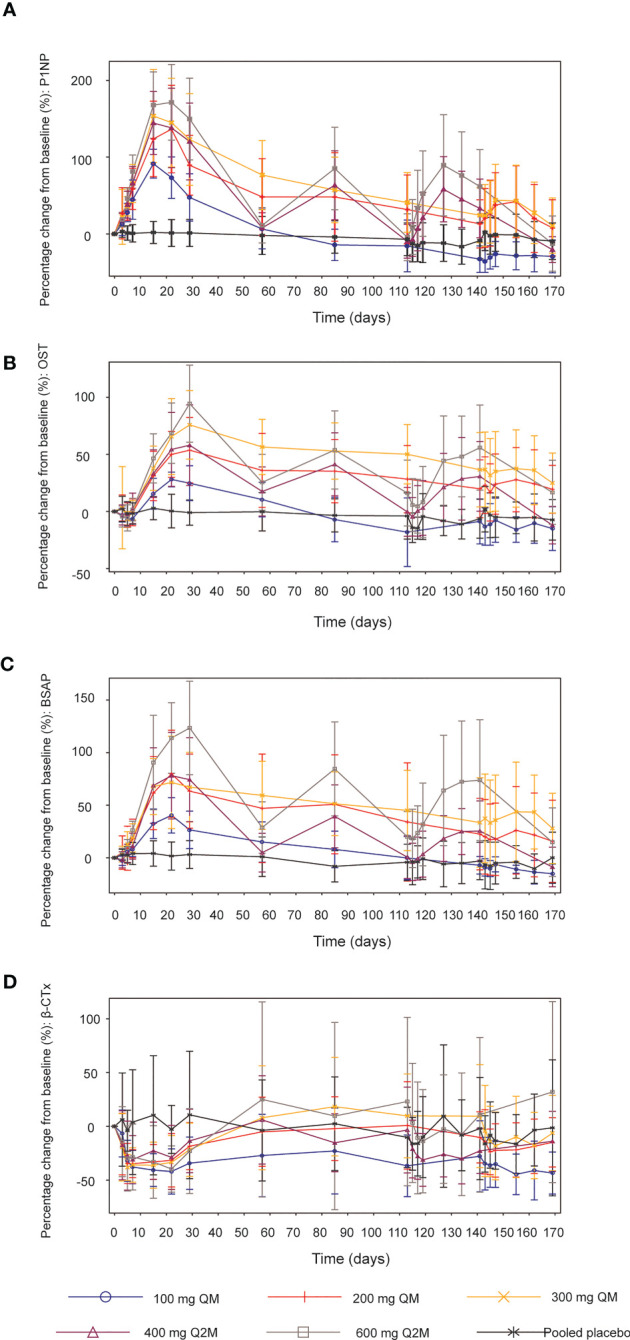
Mean (SD) percentage changes from baseline in bone turnover biomarkers. **(A)** P1NP. **(B)** OST. **(C)** BSAP. **(D)** β-CTx. SD, standard deviation; QM, once every month; Q2M, once every 2 months; P1NP, N-terminal propeptide of type 1 procollagen; OST, osteocalcin; BSAP, bone-specific alkaline phosphatase; β-CTx, β-C-telopeptide.

Mean percentage changes from baseline in the levels of the bone formation markers P1NP, BSAP, and OST and bone resorption marker β-CTx are presented in **Figure 3**.

After different dosing regimens of SHR-1222 in the order of 100, 200, and 300 mg QM; 400 and 600 mg Q2M, the serum levels of P1NP increased from the baseline and reached the peak on days 15–22. The maximum mean percentage increases in P1NP after the first administration were 91.4%, 136.4%, 153.7%, 144.5%, and 171.4% in the 100 mg QM, 200 mg QM, 300 mg QM, 400 mg Q2M and 600 mg Q2M SHR-1222 dosing group respectively, compared with 3.0% in the placebo group (**Figure 3A**). Dose-dependent increases were observed across all SHR-1222 dosing groups, and fluctuations in P1NP levels were more prominent in the Q2M dosing groups than in the QM groups. Mean P1NP concentrations gradually decreased approximately 4–8 weeks after the last dose of SHR-1222, except in the 100 mg QM group, at a consistent level below baseline, 12 weeks after the first administration. Similar trends were observed for the OST (**Figure 3B**) and BSAP (**Figure 3C**).

In addition, the bone resorption marker β-CTx significantly decreased in the SHR-1222 groups compared to the placebo group for at least the initial 4 weeks. The lowest levels were reached during days 10–22 since the first administration (-44.6%, -34.7% and -38.3% for 100 mg, 200 mg and 300 mg QM group; -31.2% and -39.7% for 400 mg and 600 mg Q2M groups, respectively; compared with -16.4% for placebo) (**Figure 3D**).

### BMD at the lumbar spine, total hip, and femoral neck

**Figure 4:**
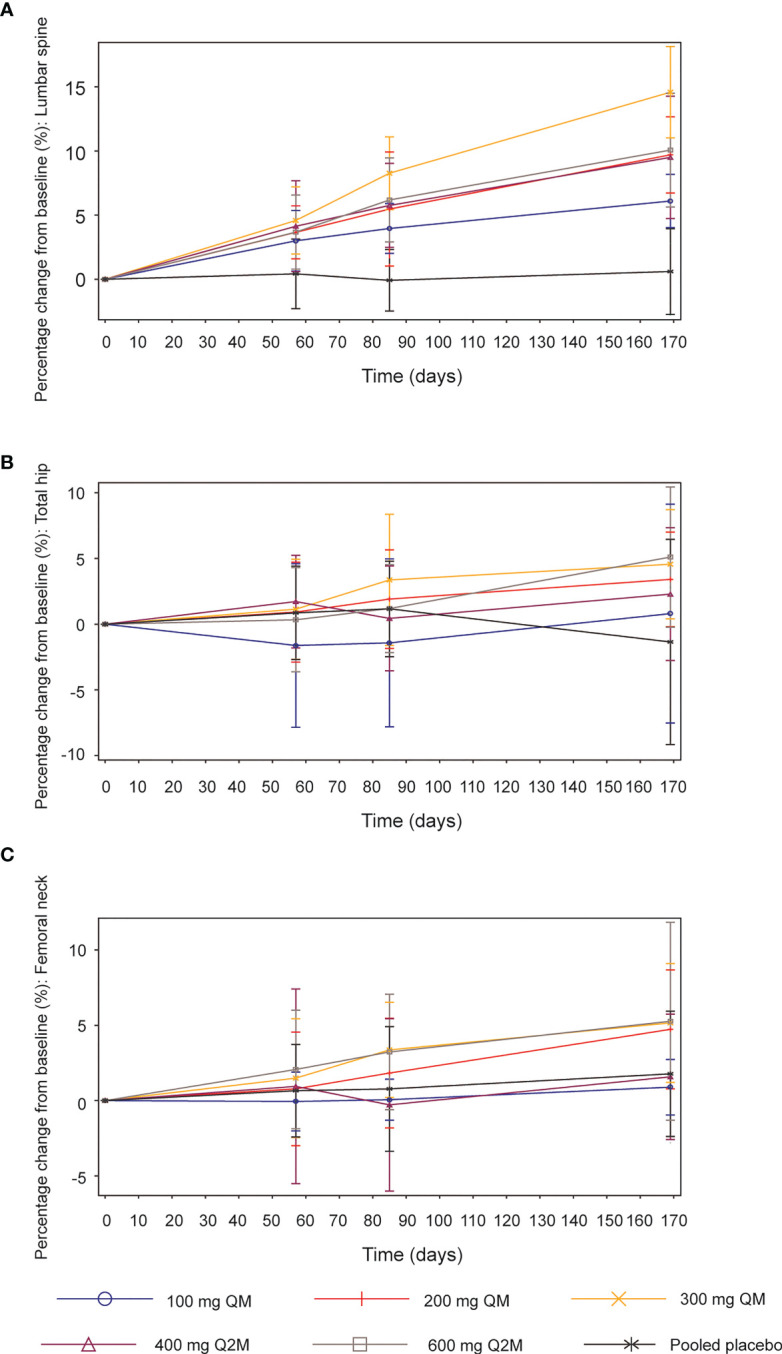
Mean (SD) percentage changes from baseline in bone mineral density (BMD). **(A)** Lumbar spine. **(B)** Total hip. **(C)** Femoral neck. SD, standard deviation; QM, once every month; Q2M, once every 2 months.

Compared with the placebo group, significant increases from baseline in BMD were observed at the lumbar spine across all SHR-1222 dosing groups 2 months after the first administration, which continued to rise throughout the subsequent treatment (**Figure 4A**). The maximum increase was detected in the 300 mg QM cohort, with percentage changes of 4.6%, 8.3%, and 14.6% on days 57, 85, and 169, respectively, compared with 0.4%, -0.1%, and 0.6% in the placebo group. The doses of 200 mg QM, 400 mg Q2M and 600 mg Q2M SHR-1222 achieved similar BMD gains in the lumbar spine at every scheduled visit. As for BMD at the total hip and femoral neck, significant increases from baseline were observed in the 200 mg QM, 300 mg QM and 600 mg Q2M SHR-1222 dosing groups 6 months after the first administration (**Figures 4B, C**). The maximum BMD gains at the total hip and femoral neck were also determined in the 300 mg QM group, with mean percentage changes of 1.1%, 3.4% and 4.6% at the total hip and 1.5%, 3.4% and 5.2% at the femoral neck on days 57, 85, and 169, respectively (compared with 0.9%, 1.2% and -1.4%; 0.7%, 0.8% and 1.8% of placebo-treated subjects). However, SHR-1222 at dosing regimens of 100 mg QM and 400 mg Q2M similarly failed to enhance BMD significantly at both the total hip and femoral neck during the entire treatment period.

### Immunogenicity

Eighty-three subjects with SHR-1222 were included in the immunogenicity analyses, among whom six (7.2%) tested positive for anti-SHR-1222 antibodies after administration (100 mg QM, n=1; 300 mg QM, n=1; 400 mg Q2M, n=1; 600 mg Q2M, n=3). However, the development of anti-SHR-1222 antibodies had no discernible effect on the main dose-standardized PK parameters (such as C_trough_/Dose, C_avg_/Dose, and C_max_/Dose) or AEs.

## Discussion

This randomized, double-blind, placebo-controlled, dose-escalation phase 1 trial is the first clinical evaluation of multiple doses of SHR-1222 for POP treatment. SHR-1222 was generally well tolerated and demonstrated an acceptable safety profile at different dosing regimens from 100 mg QM to 600 mg Q2M. The AE profile of the SHR-1222 group was comparable to that of the placebo group during the whole treatment and follow-up, except for six AEs that showed ≥ 5% higher incidences in subjects with SHR-1222 than in those with placebo. Of these, the bone-specific blood ALP level increased as expected in a dose-dependent manner, with no induction in activities of liver enzymes (such as ALT, AST, and gamma-glutamyl transferase). The increased blood ALP level may be associated with the powerful promotive effect of SHR-1222 on bone formation, which was also observed in our preclinical studies of SHR-1222 (data on file, Jiangsu Hengrui Pharmaceuticals) and is in agreement with the phase 1 trial of romosozumab (23). All six AEs were mild in severity, and none of the subjects discontinued the study owing to these AEs. On the other hand, increased serum levels of PTH, LDL, cholesterol, back pain, and arthralgia were unanticipated AEs for SHR-1222. The increases in serum PTH observed in our phase 1 trial were likely a physiological consequence of the action of sclerostin inhibitors (that is, promotion of bone formation, inhibition of bone resorption, and response to the demand for calcium reserves (24–26)), which were considered to have no clinical significance. Moreover, the increased incidence of back pain and arthralgia (all mild or moderate in severity) in subjects receiving multiple doses of SHR-1222 have also been reported in subjects receiving romosozumab (23, 27). In accordance with phase 3 trials of romosozumab for osteoporosis (15, 16), all subjects in our trial received prophylactic supplementation of calcium and vitamin D. Therefore, transient hypocalcemia or decreased blood calcium was only reported in a few subjects, which was not dose-related. Compared with those receiving placebo, subjects with SHR-1222 showed a higher incidence of increased LDL and blood cholesterol and a similar incidence of increased blood triglycerides. It has been reported that sclerostin inhibitor could increase fatty acid oxidation, reduce *de novo* fatty acid synthesis in adipocytes and promote beige adipogenesis (28, 29). The potential mechanisms underlying the effects of SHR-1222 on LDL and cholesterol levels remain unknown. However, a preclinical study proposed that cellular cholesterol homeostasis could be transiently dysregulated by blocking sclerostin and LRPs activation; their specific inhibitors elevated LDL uptake, as well as cellular lipid accumulation (30). In addition, activation of the Wnt signaling pathway may be associated with the promotion of lipid uptake (31, 32). Importantly, all these lipid-related AEs were mild, transient, and self-limiting, which did not result in withdrawal, dose interruption, or discontinuation. Therefore, the concomitant administration of SHR-1222 and drugs with a known potential to increase blood lipid levels should be closely monitored. With the exception of increased blood cholesterol, other prespecified AESIs, such as injection-site reactions, calcium and phosphate metabolic disorders, and cardiac and cerebrovascular events, showed similar incidences in the SHR-1222 and placebo groups. Furthermore, no relationship between the dose or time of unexpected AEs and AESIs was elucidated after SHR-1222 administration.

This phase 1 trial provided evidence that after multiple subcutaneous injections of SHR-1222 ranging from 100 mg QM to 600 mg Q2M, the serum exposures (C_max,ss_ and AUC_0-tau,ss_) increased with the administration schedule, showing a greater than dose-proportional manner. No marked accumulation of SHR-1222 was observed at a dose of 600 mg Q2M. After multiple doses of SHR-1222 ranging from 100 mg QM to 600 mg Q2M, the median T_max,ss_ was 4.00–5.98 d, and the mean t_1/2,ss_ was 9.59–10.5 d. The PK profiles in our trial were generally close to those reported in subjects treated with romosozumab (median T_max_, 5 d; mean t_1/2_, 12.8 d) (26), further supporting the QM dosing schedule of SHR-1222.

The dose-dependent PD responses of SHR-1222 were observed in the bone turnover biomarkers P1NP, OST, BSAP, and β-CTx, suggesting that SHR-1222 simultaneously enhances bone formation and reduces bone resorption, leading to increased BMD. The peak P1NP level increased from baseline in the SHR-1222 dosing group of 200 mg QM, which was close to that reported in a Japanese population who received romosozumab 210 mg QM (33). When comparing two studies in women with POP, we found similar rapid declines of β-CTx in all SHR-1222 dosing cohorts, as well as in romosozumab groups of 70,140 and 210 mg QM (33). Additionally, the SHR-1222 dose of 200 mg QM or 300 mg QM for 6 months improved BMD in the lumbar spine, which was close to or even exceeded the observed effects of romosozumab at a dose of 140 mg or 210 mg QM, respectively (33). The SHR-1222 300 mg QM group also showed similar BMD increases from baseline at lumbar spine and total hip to those with blosozumab at a dose of 270 mg Q2W reported in a phase 2 trial involving postmenopausal women with low BMD (T-score of -2.0 to -3.5) (20). Taken together, 300 mg QM may be selected as the recommended SHR-1222 dose in the following phase 2 trial, which presented comparable changes in PD parameters as romosozumab at its recommended dosage of 210 mg QM for postmenopausal women at high risk for fracture (17). According to the prescribing information, the treatment duration of romosozumab is 12 monthly doses because its anabolic effect wanes after 12 months (17). A similar phenomenon was observed in our phase 1 trial. The PD effect of SHR-1222 appeared to wane over the 6-month treatment, with attenuated PINP and β-CTx changes, which might be contributed to the molecular mechanisms of target therapy shared by sclerostin inhibitors.

Of the 83 subjects with SHR-1222 included in the immunogenicity analyses, six (7.2%) developed anti-SHR-1222 antibodies. Similar drug-related anti-drug antibodies were obtained after a single dose of SHR-1222 (10.0% of subjects) and romosozumab (11.1% of subjects) (22, 23). There were no discernible effects of anti-SHR-1222 antibodies on PKs, PDs, or AEs. Notably, considering that SHR-1222 neutralizing antibodies were not determined in our phase 1 trial, the interpretation of the current findings requires further investigation.

In conclusion, in this dose-escalation phase 1 trial, multiple doses of SHR-1222 were safe and well-tolerated ranging from 100 mg QM to 600 mg Q2M. The serum SHR-1222 exposure (C_max,ss_ and AUC_0-tau,ss_) increased in a greater than dose-proportional manner. PD analyses indicated that multiple doses of SHR-1222 promoted bone formation, decreased bone absorption, and consequently resulted in rapid and prominent improvement in BMD. These findings further support SHR-1222 as a potential alternative agent for the treatment of POP, as well as other conditions that may benefit from promoting bone formation, especially in those at high or imminent fracture risk.

## Data availability statement

The original contributions presented in the study are included in the article/supplementary material. Further inquiries can be directed to the corresponding author.

## Ethics statement

The studies involving human participants were reviewed and approved by the Ethics Committee of the Second Xiangya Hospital of Central South University and all other participating centers. The patients/participants provided their written informed consent to participate in this study.

## Author contributions

Conceptualization: ZD, RZ, ZS, HC, and ZGZ; Data curation: ZD, RZ, ZS, GQ, XL, QQ, CLS, LL, PJ, GY, YC, DP, CZ, LW, JS, QZ, ZZ, XY, PF, QY, LY, XF, WLiu, BW, RC, XW, YX, and ZGZ; Formal analysis: WW; Supervision: ZD, RZ, ZS, HC, and ZGZ; Project administration: ZD, RZ, ZS, CS, KS, WW, WLu, HC, and ZGZ; Writing – original draft: ZD, RZ, ZS, HC, and ZGZ; Investigation: All authors; Methodology: All authors; Writing – review & editing: All authors. All authors contributed to the article and approved the submitted version.
